# Diminution of Hepatic Response to 7, 12-dimethylbenz(α)anthracene by Ethyl Acetate Fraction of *Acacia catechu* Willd. through Modulation of Xenobiotic and Anti-Oxidative Enzymes in Rats

**DOI:** 10.1371/journal.pone.0090083

**Published:** 2014-02-27

**Authors:** Rakesh Kumar, Rajbir Kaur, Amrit Pal Singh, Saroj Arora

**Affiliations:** 1 Department of Botanical and Environmental Sciences, Guru Nanak Dev University, Amritsar, India; 2 Department of Pharmaceutical Sciences, Guru Nanak Dev University, Amritsar, India; Universidade Federal do Rio de Janeiro, Brazil

## Abstract

**Background:**

Liver is the primary metabolizing site of body and is prone to damage by exogenous as well as endogenous intoxicants. Polycyclic aromatic hydrocarbons such as 7, 12- dimethylbenz(α)anthracene (DMBA) is an exogenous hepatotoxin, which is well known for modulating phase I, II and anti-oxidative enzymes of liver. Plants contain plethora of polyphenolic compounds which can reverse the damaging effect of various xenobiotics. The present study investigated protective role of the ethyl acetate fraction of *Acacia catechu* Willd. (EAF) against DMBA induced alteration in hepatic metabolizing and anti-oxidative enzymes in rats.

**Methodology and Principal Findings:**

The rats were subjected to hepatic damage by treating with DMBA for 7 weeks on alternative days and treatment schedule was terminated at the end of 14 weeks. The rats were euthanized at the end of protocol and livers were homogenized. The liver homogenates were used to analyse phase I (NADPH-cytochrome P450 reducatse, NADH-cytochrome b5 reductase, cytochrome P420, cytochrome b5), phase II (glutathione-S-transferase, DT diaphorase and γ-Glutamyl transpeptidase) and antioxidative enzymes (catalase, superoxide dismutase, ascorbate peroxidase, glutathione reductase, guiacol peroxidase and lactate dehydrogenase). Furthermore, other oxidative stress parameters (thiobarbituric acid reactive substances, lipid hydroperoxides and conjugated dienes and reduced glutathione) and liver marker enzymes (serum glutamic oxaloacetic transaminase, serum glutamic pyruvic transaminase and alkaline phosphatase) were also studied. The DMBA induced significant changes in activity of hepatic enzymes that was reversed by treatment with three dose levels of EAF.

**Conclusion:**

It is concluded that EAF affords hepato-protection against DMBA in rats through modulation of phase I, II and anti-oxidative enzymes.

## Introduction

Liver is the most important organ of body involved in numerous detoxification processes, synthesis of plasma proteins and plays vital role in digestion. Being primary metabolic site, it is prone to damage mediated by exogenously administered or endogenous intoxicants released during metabolism of various substances in human body. These agents cause severe damage to liver thereby resulting in hepatitis, cholestatic, fibrosis, granulomas and vascular lesions [1], [2], [3], [4], [5]. Various hepato-toxins act by initiating secondary and tertiary changes in the gene expression, eliciting immune mediated allergic responses and modulating activities of xenobiotic and anti-oxidative enzymes [6].

Polycyclic aromatic hydrocarbons such as 7, 12- dimethylbenz(α)anthracene (DMBA) leads to hepatotoxicity, carcionogenicity as well as alteration of phase I and II enzymes involved in metabolic processes in liver. The methyl-hydroxylated derivatives, phenolic derivatives as well as dihydrodihydroxy derivatives of DMBA specifically target adenine residues of DNA inducing mutations and xenobiotic enzyme alteration in liver [7], [8], [9]. It is also known to suppress both cell mediated and humoral immune responses in mice and related species [10]. Many attempts have been made in past to treat DMBA induced hepatic alterations in rats using extracts prepared from medicinal plants [8].

*Acacia catechu* Willd. (Family: Mimosaceae) also known as khadira, khair, karingali and katha is an important medicinal plant found in relatively drier regions of India [11], [12]. There are umpteen references available in Ayurveda projecting it as a valuable tree having numerous medicinal properties. Its antipyretic, anti-diarrhoel, hepato-protective and hypo-glycemic properties have been reported [13]. Many polyphenols like catechin, rutin, isorhamnetin and epicatechin have been isolated which could be the reason for its varied medicinal value [12], [14]. The present study has been designed to investigate protective role of the ethyl acetate fraction of *Acacia catechu* Willd. against DMBA induced alteration in hepatic metabolizing and antioxidant enzymes in rats.

## Materials and Methods

### Chemicals

DMBA (7, 12- dimethylbenz(α)anthracene), Indole 3 carbinol (I3C), 5-5′dithiobisnitrobenzoic acid (DTNB) sodium dithionate and malondialdehyde were obtained from Sigma-aldrich, Banglore, India. Xylenol orange, ammonium ferrous sulfate, cyclohexane, 2,6-dichlorophenol-indophenol (DCPIP), reduced nicotinamide adenine dinucleotide (NADH), reduced nicotinamide adenine dinucleotide phosphate (NADPH), bovine serum albumin (BSA), potassium ferricyanide, 1-chloro-2,4-dinitrobenzene (CDNB), glycylglycine, reduced glutathione (GSH), oxidized glutathione (GSSG), ascorbic acid, guiacol, triton X, nitroblue tetrazolium chloride (NBT), hydroxylamine hydrochloride, pyruvate were obtained from Himedia Laboratories, Mumbai, India. All other reagents used in the present study were of analytical grade.

### Ethical statement

The rats were housed in central animal house of Guru Nanak Dev University (GNDU), Amritsar as per the guidelines of committee for the purpose of control and supervision of experiments on animals (CPCSEA), Ministry of Environment and Forests, Government of India. The study was approved by Institutional Animal Ethics Committee of GNDU, Amritsar (671/BT dated 20th May 2011).

### Preparation of extract

The leaves of *Acacia catechu* Willd. were harvested from tree growing in the GNDU campus, Amritsar. Taxonomic identification was made by herbarium of Department of Botanical and Environmental Sciences, GNDU, Amritsar. The leaves of plant were thoroughly washed with water, dried at room temperature and ground to fine powder. The powdered leaves were extracted with 80% methanol and further fractionated using different solvents viz. hexane, chloroform, ethyl acetate to obtain hexane, chloroform and ethyl acetate fractions. The supernatant was filtered using Whatman No. 1 sheet, pooled and concentrated using vacuum rotary evaporator (Buchi Rotavapor R-210). The concentrated solutions were then lyophilized to get the dried form of fractions. For present study, the polyphenolic rich ethyl acetate fraction of *A catechu* (EAF) was used to evaluate its protective potential in 7, 12-dimethylbenz(α)anthracene induced hepatic damage in rats.

### Ultra High Pressure Liquid Chromatographic analysis of EAF

For UHPLC analysis EAF (10 mg) was dissolved in 1 ml methanol (HPLC grade) and filtered through 0.22 µm syringe filter (PALL Life Sciences). Sample was analyzed on Shimadzu UHPLC Nexera system (Shimadzu, MA, USA), provided with a photodiode array (PDA) detector using C_18_ column (150 mm×4.6 mm, i.d. 5 µm). The column temperature was maintained at 25°C and gradient mobile phase consisting of 0.1% acetic acid aqueous as solution A and Methanol as solution B was used. The gradient elution is: 0–1 min, 30% B; 1–10 min, 65% B; 10–14 min, 80% B; 14–16 min, 80% A, 16–17 min: 40% B, 17–20 min: 35% B and 20–21 min: 30% B. The flow rate was set as 1 ml/min and the injection volume was 5 µl. Quantification of peaks was also done using software provided with Shimadzu UHPLC Nexera system.

### Treatment of rats and preparation of liver homogenate

**Figure 1:**
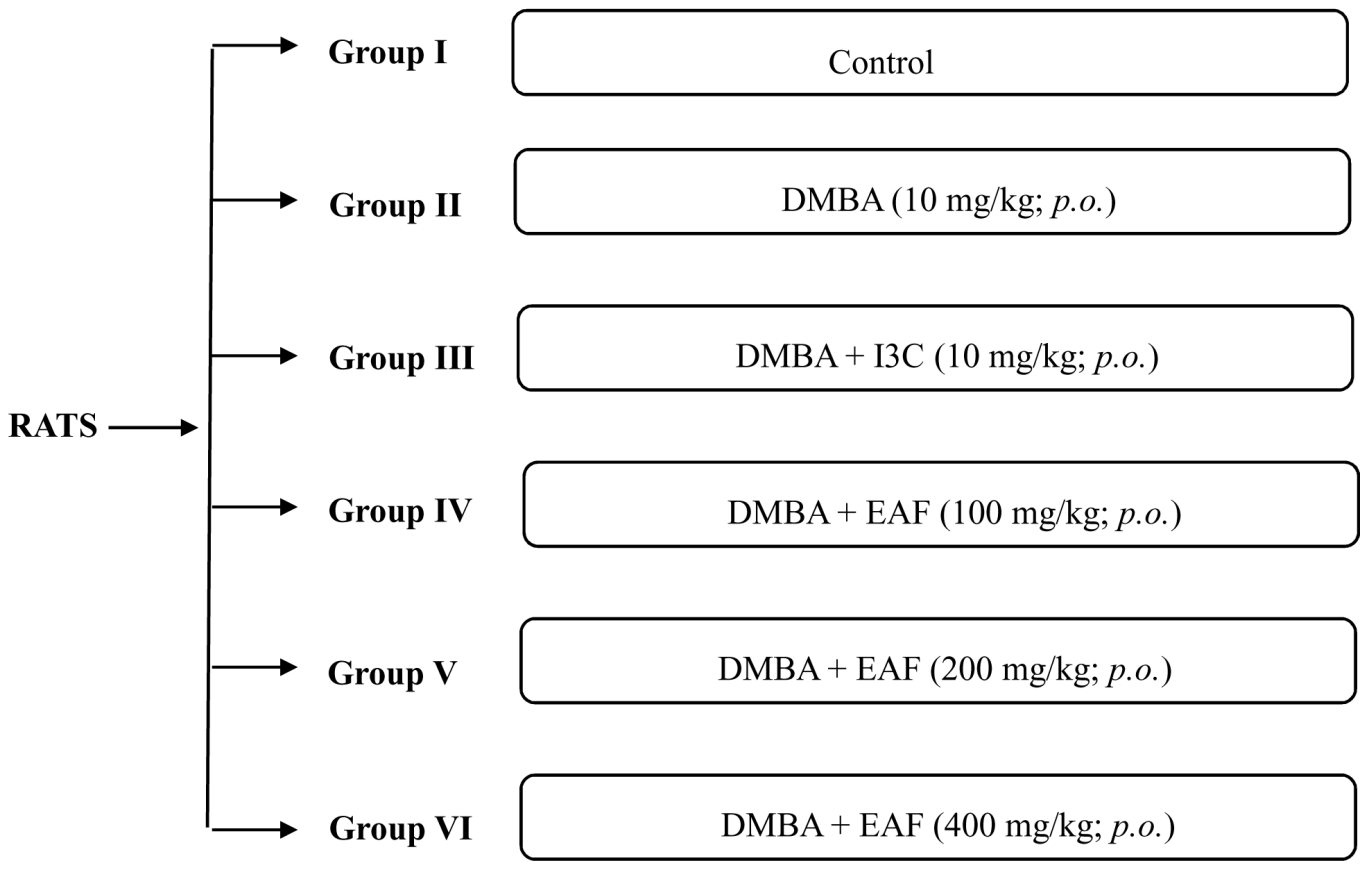
Showing treatment groups, where I3C  =  Indole 3 carbinol; EAF  =  Ethyl acetate fraction; DMBA  =  7, 12- dimethylbenz(α)anthracene.

Female sprague dawley rats (6–8 weeks old) with body weight of 150–200 g were used in the present study. The rats were divided into 6 groups having 4 in each group. Different concentrations of DMBA (10 mg/kg), Indole-3-Carbinol (I3C 10 mg/kg) and EAF (100, 200, 400 mg/kg) were made in peanut oil and given to rats *via* oral route on alternative days (figure 1). The treatments were given for seven weeks and the experiment was terminated at the end of 14 weeks. Rats were euthanized at the end of protocol. The serum and tissue samples were stored at −20°C for further analysis.

The liver samples were homogenized in ice cold 0.15 M Tris-KCl buffer to yield 10% (w/v) homogenate. 0.5 ml of this homogenate was precipitated with 5% trichloroacetic acid, centrifuged at 2000 rpm and the supernatant was used for the estimation of reduced glutathione content. The homogenate was used to analyse the activity of phase I and II enzymes such as NADPH-cytochrome P450 reducatse, NADH-cytochrome b5 reductase, cytochrome P420, cytochrome b5 and glutathione-S-transferase (GST), DT diaphorase (DTD) and γ-Glutamyl transpeptidase (GGT), respectively. The activity of various antioxidant enzymes viz., catalase (CAT), superoxide dismutase (SOD), ascorbate peroxidase (APOX), glutathione reductase (GR), guiacol peroxidase (GPOX) and lactate dehydrogenase (LDH) was also estimated in liver homogenate. Furthermore, the homogenate estimation of lipid peroxides in terms of malondialdehyde, lipid hydroperoxide content and conjugated dienes was done. The level of serum glutamic oxaloacetic transaminase (SGOT), serum glutamic pyruvic transaminase (SGPT) and alkaline phosphatase (ALP) was also determined in serum.

### Effect of pharmacological interventions on phase I enzymes in liver

NADPH-cytochrome P450 reductase (E.C.1.6.2.4) activity was determined following the method described by Omura and Takasue [15] and activity of NADH-cytochrome b5 reductase (E.C.1.6.2.2) was assayed according to the method of Mihara and Sato [16] with slight modifications. For NADPH-cytochrome P450 reductase, 0.3 M phosphate buffer (pH = 7.5) was added to 0.1 mM NADPH followed by 0.2 mM potassium ferricyanide and tissue homogenate, such that final volume makes upto 1 ml. Absorbance was taken at 340 nm. The enzyme activity was calculated using extinction coefficient 6.22 mM^−1^cm^−1^. The activity of NADH-cytochrome b5 reductase was calculated using reaction mixture comprised of 0.1 M potassium phosphate buffer (pH = 7.5), 0.1 mM NADH, 1 mM potassium ferricyanide and homogenate in a final volume of 1 ml. Absorbance was read at 420 nm. The enzyme activity was calculated using extinction coefficient of 1.02 mM^−1^cm^−1^.

Cytochrome P420 content was determined using the carbon monoxide difference spectra as obtained by following the methodology of Choi et al. [17]. Cytochrome P420 activity was determined from the change in absorbance at 420 nm and 490 nm using an absorption coefficient of 111 mM^−1^cm^−1^. The spectral difference between 424 nm and 409 nm of oxidized and NADH reduced homogenate using an absorption coefficient of 185 mM^−1^cm^−1^ led to the estimation of cytochrome b5 content [18].

### Effect of pharmacological interventions on phase II enzymes in liver

Glutathione-S-transferase (E.C. 2.5.1.18) activity was determined spectrophotometrically as described by Habig et al. [19]. The reaction was started by adding 0.1 ml of tissue homogenate to reaction mixture (0.1 M phosphate buffer (pH = 6.5), 30 mM CDNB and 30 mM reduced glutathione), followed by incubation at 37°C for 3 minutes. The absorbance was read at 340 nm for 3 minutes. The GST activity was calculated using an extinction coefficient of 9.6 mM^−1^cm^−1^.

NAD(P)H:quinone oxidoreducatse (EC 1.6.99.2) also known as DT-diaphorase was measured according to the method of Ernster [20] with slight modifications. The reaction mixture comprising of 0.05 M Tris Buffer (pH = 7.5–8.0), NADH (0.3 mM), DCPIP (0.4 mM), BSA (0.7%) was mixed with homogenate and the absorbance was measured at 600 nm. The activity was calculated using an extinction coefficient 21 mM^−1^cm^−1^.

γ- Glutamyltranspeptidase (EC 2.3.2.2) enzyme has an important role in the detection of liver related disorders including tumors. The enzyme activity was determined following method of Szasz [21]. To 1 ml of working reagent (prepared by mixing 5 mM L- γ -glutamyl-4-nitroanilide in 0.1 N HCl (R1) and 0.1 M of glycylglycine in 0.1 M Tris Buffer (pH = 8.6) (R2) in the ratio of 1∶4) was added 0.1 ml of homogenate followed by incubation for 1 minute. The absorbance was measured at 405 nm. The activity of GGT was calculated by multiplying mean change in absorbance per minute with a factor (1158).

Effect of pharmacological interventions on anti-oxidant enzymes in liver

Catalase (E.C. 1.11.1.6) and superoxide dismutase (EC 1.15.1.1) was assayed following the method given by Aebi [22] and Kono [23] respectively. Ascorbate peroxidase (EC 1.11.1.11) is involved in the detoxification of peroxides such as hydrogen peroxide using ascorbate as a substrate. The enzyme activity was determined by the method of Asada [24]. Glutathione reductase (EC 1.8.1.7) was determined by the method of Carlberg and Mannervik [25]. Guiacol peroxidase (EC 1.11.1.7) was determined by the method as given by Putter [26]. Lactate dehydrogenase (EC 1.1.1.27, L-lactate: NAD^+^ oxidoreductase) activity was determined on the basis of rate of oxidation of pyruvate at 340 nm by NADH following the method of Kuznetsov and Gnaiger [27].

### Effect of pharmacological interventions on other oxidative stress parameters in liver homogenate

Lipid peroxidation was determined in terms of the formation of thiobarbituric acid reactive substances (TBARS), lipid hydroperoxides and conjugated dienes following the method of Devasagayam et al. [28], Jiang et al. [29] and Devasagayam et al. [28] respectively with slight modifications. The amount of reduced glutathione in the tissue was determined using the method as described by Anderson [30]. For analysis, TCA (5%) was added to the tissue homogenate and 0.1 ml of later was added to 0.2 M phosphate buffer (pH = 8) and mixed with 0.6 mM of DTNB. The reaction mixture was incubated for 10 minutes. The absorbance was noted at 412 nm.

### Effect of pharmacological interventions on hepatic enzyme activity

Estimation of liver tissue marker enzymes like serum glutamic oxaloacetic transaminase (SGOT), serum glutamic pyruvic transaminase (SGPT) and alkaline phosphatase (ALP) were estimated using auto packed kits procured from Delta Lab, Sindhudurg, India.

### Statistical analysis

The experimental data were expressed as mean ± SE. One way analysis of variance (ANOVA) and Tukey's HSD post hoc test were carried out to determine significant differences between the means at p≤0.05.

## Results

### Ultra High Pressure Liquid Chromatographic analysis of EAF fraction

**Figure 2:**
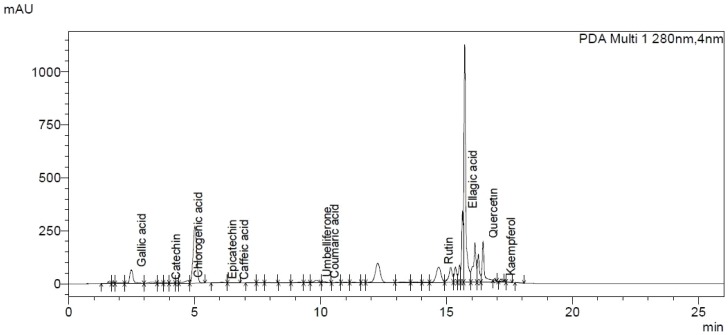
UHPLC chromatograph of EAF.

UHPLC analysis showed the abundance of polyphenolic compounds (Table 1). Ellagic acid, rutin and quercetin were the major compounds present in EAF. Other polyphenols were also detected but in minor amounts viz., gallic acid, catechin, chlorogenic acid, epicatechin, caffeic acid, umbelliferone, coumaric acid and kaempferol (Figure 2).

**Table 1:** Percentage of polyphenolic compounds in EAF as detected by UHPLC analysis.

S.No	Compounds	Percentage
1	Ellagic acid	3.113
2	Rutin	2.500
3	Quercetin	1.838
4	Gallic acid	0.446
5	Umbelliferone	0.384
6	Kaempferol	0.251
7	Chlorogenic acid	0.227
8	Catechin	0.135
9	Epicatechin	0.126
10	Coumaric acid	0.027
11	Caffeic acid	0.009

### Effect of pharmacological interventions on phase I enzymes in liver

**Figure 3:**
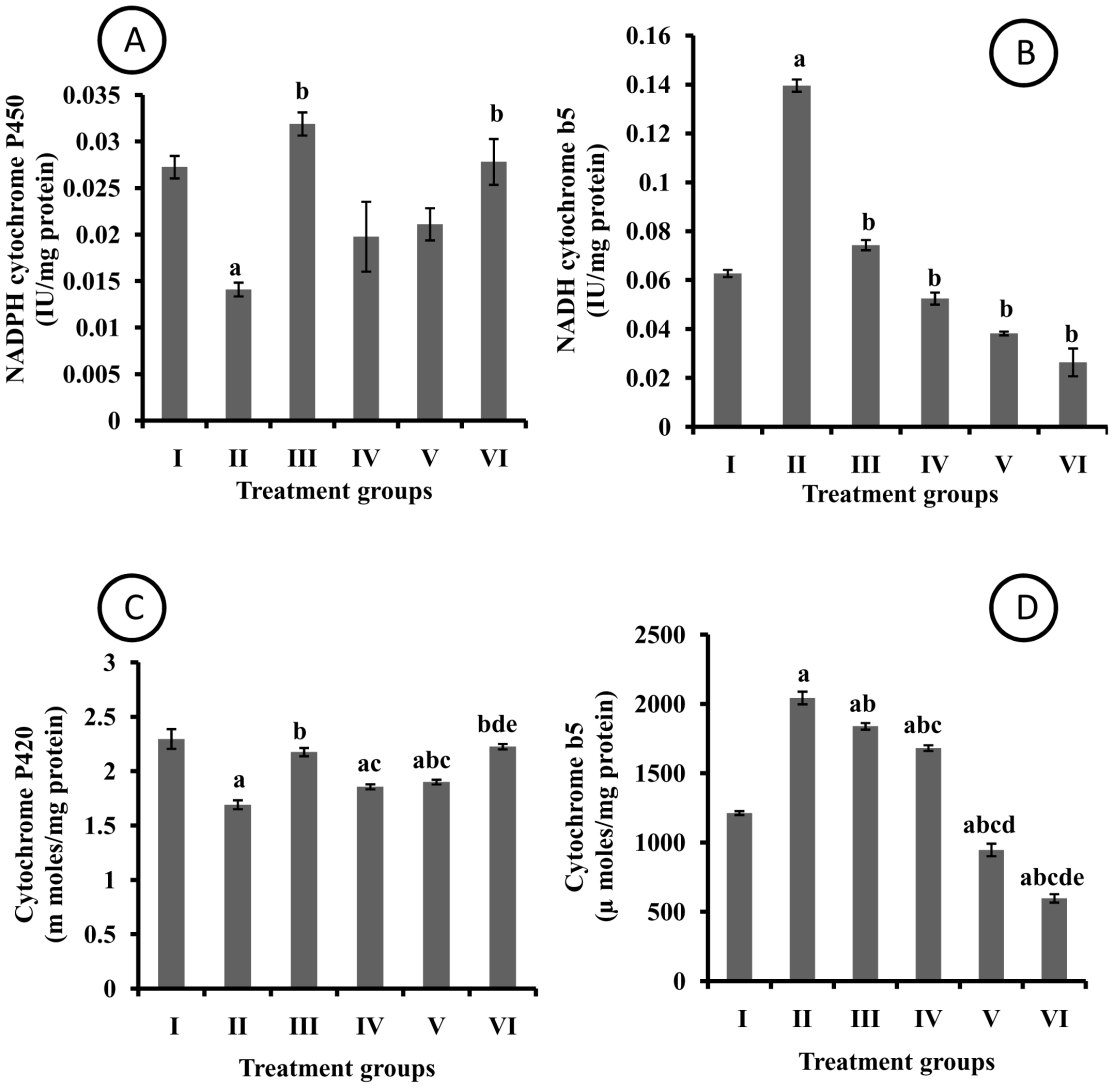
Effect of pharmacological interventions on phase I enzymes in liver (A) NADPH cytochrome P450 reductase (B) NADH cytochrome b5 reductase (C) Cytochrome P420 content (D) Cytochrome b5 content. a  =  p≤0.05 vs control group; b  =  p≤0.05 vs DMBA group; c  =  p≤0.05 vs I3C group; d  =  p≤0.05 vs EAF 100 group; e  =  p≤0.05 vs EAF 200 group.

A significant decrease in NADPH cytochrome P450 enzyme activity was observed in liver homogenate of DMBA treated rats as compared to control group. The treatment with I3C standard abolished DMBA induced decrease in enzyme activity. The treatment with EAF 100 and 200 did not afford any protection against DMBA induced reduction in enzyme activity however; EAF 400 group witnessed significant protection (Figure 3A). A significant increase in NADH cytochrome b5 enzyme activity was observed in liver homogenate of DMBA treated rats as compared to control group. The treatment with I3C standard as well as three dose levels of EAF afforded significant protection against DMBA induced increase in NADH cytochrome b5 activity (Figure 3B).

The hepatic cytochrome P420 enzyme activity was significantly reduced in DMBA treated rats as compared to control rats. The treatment with I3C, EAF 200 and 400 significantly attenuated DMBA induced decrease in hepatic enzyme activity. Moreover, the protection afforded by EAF 400 was significantly higher than EAF 200 group (Figure 3C).

A significant increase in cytochrome b5 enzyme was observed in liver homogenate of DMBA treated rats as compared to control group that was reversed by I3C treatment in rats. The EAF 100, 200 and 400 groups witnessed significant protection in a dose dependent manner (Figure 3D).

### Effect of pharmacological interventions on phase II enzymes in liver

**Figure 4:**
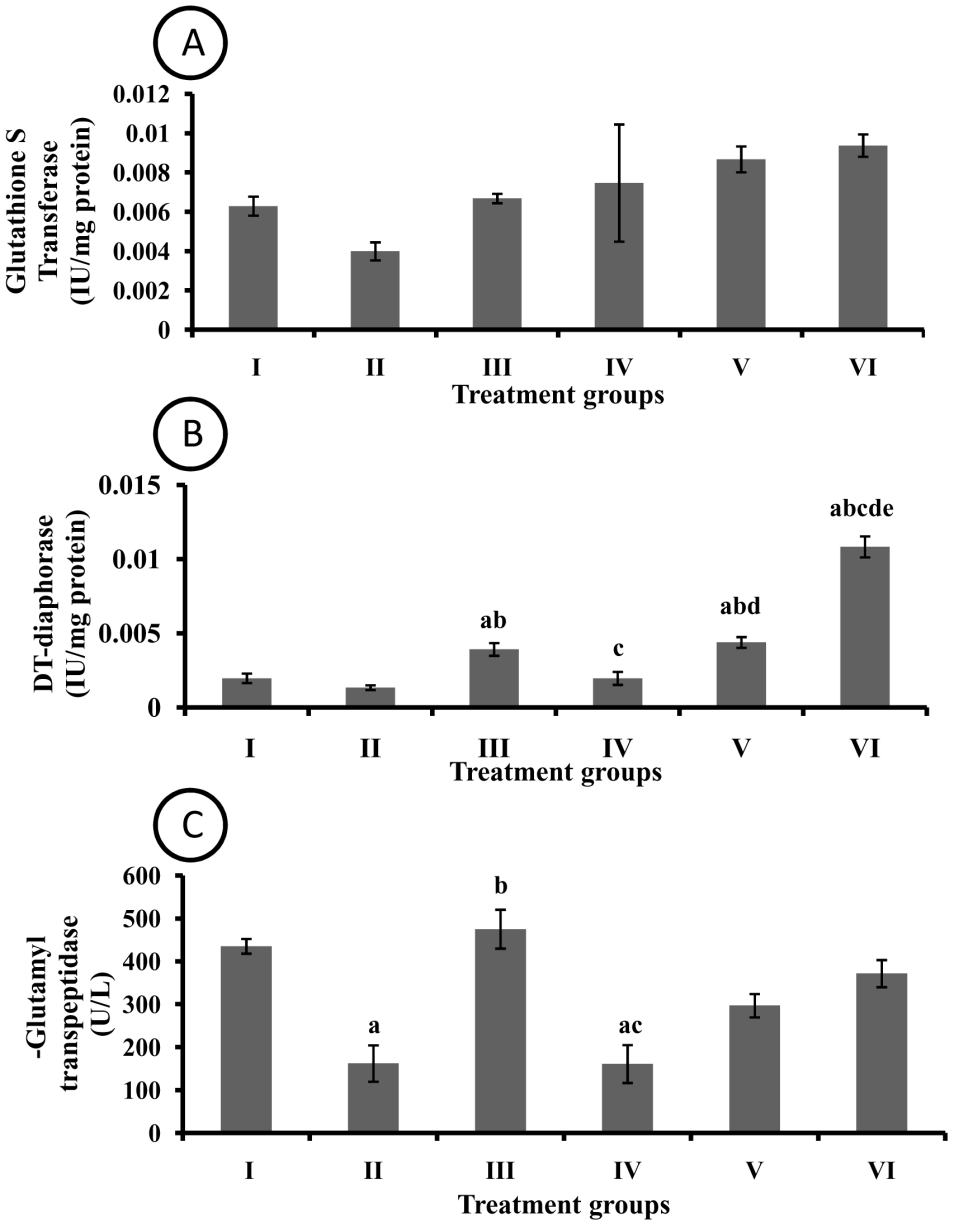
Effect of pharmacological interventions on phase II enzymes in liver (A) Glutathione-S-transferase (B) DT-diaphorase (C) γ-Glutamyl transpeptidase. a  =  p≤0.05 vs control group; b  =  p≤0.05 vs DMBA group; c  =  p≤0.05 vs I3C group; d  =  p≤0.05 vs EAF 100 group; e  =  p≤0.05 vs EAF 200 group.

No significant change in activity of GST was observed among various groups employed in the present study (Figure 4A). No significant difference in DTD activity was observed between control and DMBA treated rats. The I3C treatment significantly increased DTD activity as compared to control and DMBA treated group. Moreover, EAF treatment significantly increased DTD activity in a dose dependent manner (Figure 4B). The GGT activity was significantly decreased in DMBA treated group as compared to control group. The I3C treatment significantly attenuated DMBA induced decrease in hepatic GGT activity however; the EAF fraction had no significant effect on GGT activity (Figure 4C).

### Effect of pharmacological interventions on anti-oxidant enzymes in liver

**Figure 5:**
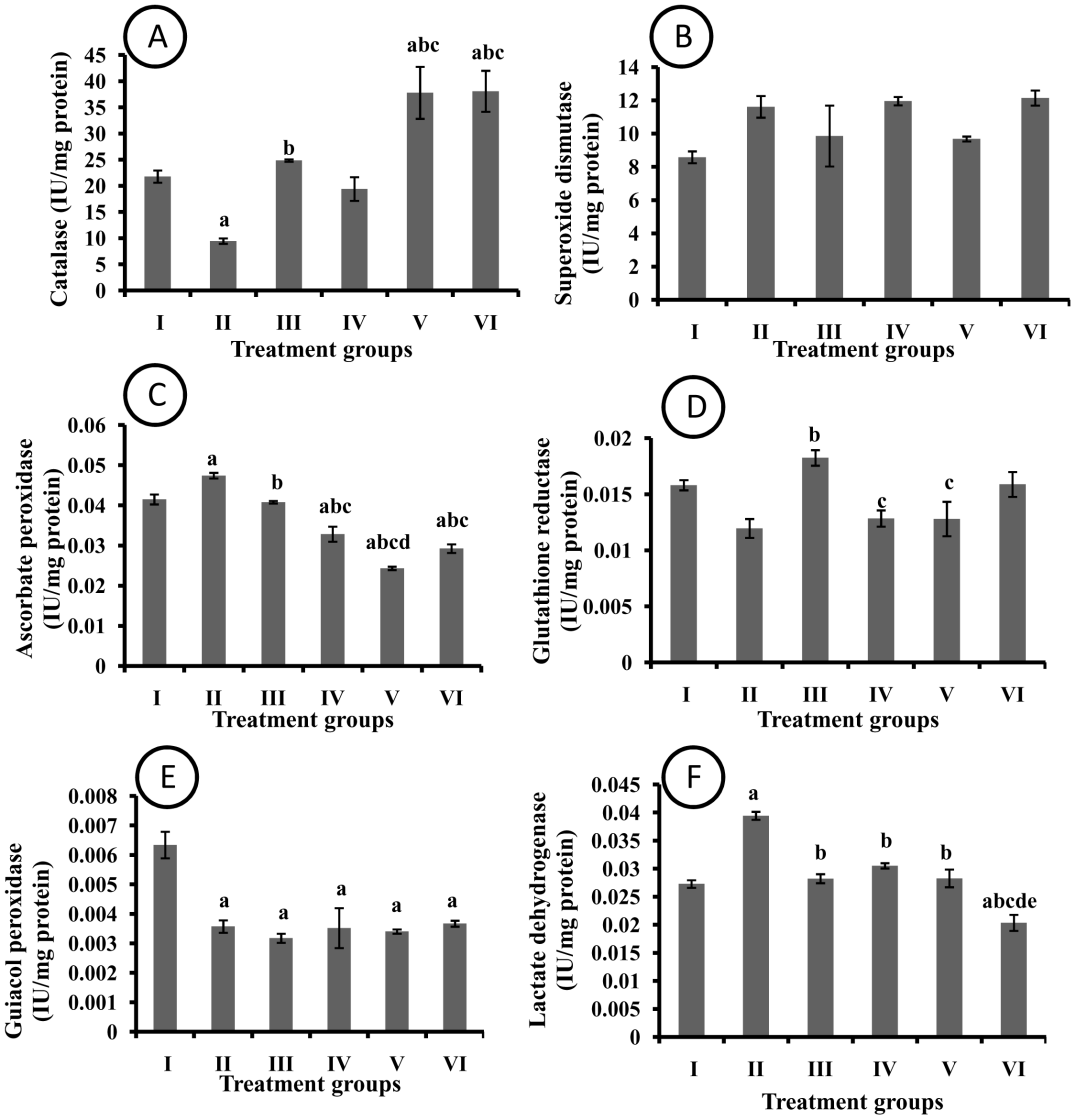
Effect of pharmacological interventions on anti-oxidative enzymes in liver (A) Catalase (B) Superoxide dismutase (C) Ascorbate peroxidase (D) Glutathione reductase (E) Guiacol peroxidase (F) Lactate dehydrogenase. a  =  p≤0.05 vs control group; b  =  p≤0.05 vs DMBA group; c  =  p≤0.05 vs I3C group; d  =  p≤0.05 vs EAF 100 group; e  =  p≤0.05 vs EAF 200 group.

A significant reduction in CAT activity was observed in DMBA treated group as compared to control group that was abolished by I3C treatment in rats. The protection observed in EAF 200 and 400 groups was significant as compared to DMBA as well as I3C treated group (Figure 5A). No significant change was observed in SOD activity among various groups employed in the present study (Figure 5B). A significant increase in hepatic APOX activity was observed in DMBA treated rats as compared to control group. The treatment with I3C significantly abolished DMBA induced increase in APOX activity. Moreover, the EAF treatment witnessed significant protection as compared to DMBA as well as I3C treated group (Figure 5C). The GR activity increased significantly in I3C treated rats as compared to DMBA treated rats. The EAF doses observed no significant change in GR activity as compared to DMBA treated rats (Figure 5D). A significant decrease in GPOX activity was observed in various groups as compared to control group however, no significant difference in GPOX activity was observed among other groups employed in the present study (Figure 5E). The LDH activity was significantly increased in DMBA group as compared to control group. The treatment with I3C and three doses of EAF significantly attenuated DMBA induced increase in lactate dehydrogenase activity. Moreover, the EAF 400 group observed significant protection as compared to I3C, EAF 100 and 200 groups, respectively (Figure 5F).

### Effect of pharmacological interventions on other oxidative stress parameters in liver homogenate

The DMBA group observed significant increase in lipid peroxides measured in terms of TBARS as compared to control group (Table 2). The treatment with I3C and EAF afforded protection against DMBA induced generation of lipid peroxides in hepatic tissues of rats. A significant increase in lipid hydroperoxides was observed in DMBA treated rats as compared to control group that was significantly attenuated with I3C treatment. Moreover, the EAF treatment markedly attenuated lipid hydroperoxides in a dose independent manner. The DMBA group witnessed significant rise in the level of conjugated dienes as compared to control group. The I3C and EAF treated groups witnessed significant reduction in conjugated dienes. Moreover, the reduction in EAF 200 and 400 treated groups was significantly less than I3C and EAF 100 treated group. A significant reduction in GSH was observed in DMBA treated rats as compared to control group. The I3C treatment reversed DMBA induced reduction in glutathione level. The EAF treatment did not observe any effect on GSH level in rats.

**Table 2:** Shows TBARS, Lipid Hydroperoxide, Conjugated diene and Reduced glutathione content.

Groups	TBARS (µmoles MDA equivalent/g of tissue) ± SE	Lipid Hydroperoxide (mM H_2_O_2_ equivalents/g of tissue ) ± SE	Conjugated dienes (n moles/mg protein) ± SE	Reduced Glutathione content (µ moles of SH content/g of tissue) ± SE
**I**	29.71±3.580	4.91±0.0757	190.33±13.613	296.25±6.967
**II**	118.81±16.213^a^	7.64±0.352^a^	340.94±28.242^a^	242.77±10.056^a^
**III**	28.31±2.151^b^	5.32±0.173^b^	188.72±13.212^b^	281.01±4.517^b^
**IV**	31.8±0.179^b^	4.87±0.639^b^	191.29±9.893^b^	250.97±6.269^ac^
**V**	26.40±2.937^b^	4.19±0.455^b^	94.62±16.303^abcd^	255.82±7.526^a^
**VI**	21.33±1.83^b^	5.83±0.292^b^	69.11±8.845^abcd^	265.64±2.863^a^

### Effect of pharmacological interventions on hepatic enzyme activity

**Figure 6:**
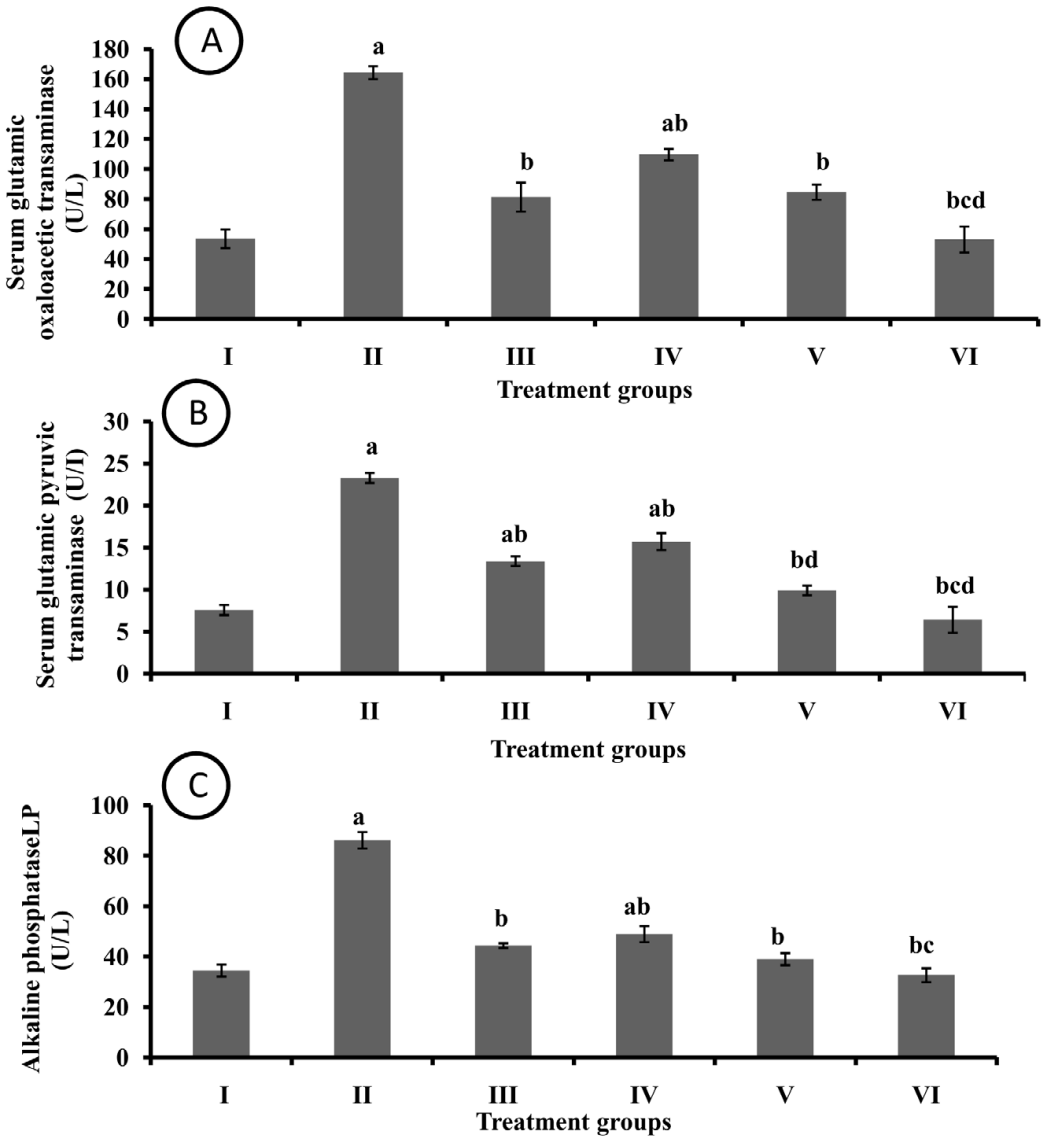
Effect of pharmacological interventions on hepatic enzyme activity (A) Serum glutamic oxaloacetic transaminase (B) Serum glutamic pyruvic transaminase (C) Alkaline phosphatase. a  =  p≤0.05 vs control group; b  =  p≤0.05 vs DMBA group; c  =  p≤0.05 vs I3C group; d  =  p≤0.05 vs EAF 100 group.

A significant increase in serum SGOT activity was observed in DMBA group as compared to control group. The I3C treatment significantly attenuated DMBA induced increase in SGOT activity. The three doses of EAF afforded significant protection with maximum effect observed in EAF 400 group (Figure 6A). Similar pattern of results was observed in serum SGPT activity (Figure 6B). The DMBA treated rats observed significant increase in ALP activity as compared to control group. The I3C and EAF treatment (100, 200 and 400) significantly abolished DMBA induced increase in serum ALP levels (Figure 6C).

## Discussion

Modern medicinal system relies heavily on synthetic chemicals being used as drugs, but these unnatural synthetic drugs often pose serious side effects [31], [32], [33]. Natural phytochemicals thus can provide bioactive phytoconstituents that could substitute synthetic chemicals and reduce the risk of side effects [34], [35]. The present study explores the protective role of EAF against DMBA induced changes in hepatic xenobiotic and oxidative enzymes in rats.

Phase I biotransformation reactions increase the polarity of xenobiotics either by adding or exposing functional groups and thereby facilitating their excretion from body. Oxidation, reduction and hydrolysis are three important reactions through which phase I metabolism operates [36]. During drug metabolism, three-fourths of total enzymatic reactions are catalyzed by P450s [37]. An increase in the NADPH cytochrome p450 and cytochrome p420 was observed in all treatment groups as compared to DMBA treated rats. The NADH cytochrome b5 and cytochrome b5 showed decreasing trend as compared to DMBA treated rats. Elevated level of NADH cytochrome b5 and cytochrome b5 in group II rats might be due to the role of these enzymes in metabolizing DMBA.

Phase II drug metabolism involves biotransformation of phase (I) products, making them pharmacologically less active and more excretable form [38]. All the phase II enzymes studied viz., GST, DTD and GGT observed increase in all EAF treated rats. DMBA treatment decreased the expression of phase II enzymes as compared to control group of rats. GST catalyzes detoxification of xenobiotics and reduction of organic hydroperoxides by conjugating glutathione to them [39]. An increase in GST activity might be due to increased lipid hydroperoxides as was observed in EAF treated groups. DTD is known for its wide array of functions; still it is mainly responsible for maintaining reduced state of ubiquinines thus providing protection against oxidative damage [40]. A dose dependent increase of DTD activity in EAF treated rats might be due to its hepatoprotective potential exerted by nullifying oxidative injury and restoring antioxidant systeme

Reactive oxygen species (ROS) have been known to be involved in various pathophysiological conditions [41], [42], [43]. The DMBA treatment induced significant oxidative stress in rats. The treatment with EAF counteracted oxidative stress by increasing the activity of catalase and other anti-oxidative enzymes. These results are in accordance with study of Salama et al. [33] where rise in the level of SOD and CAT was observed after treatment of ethanolic extract of *Curcuma longa* in thioacetamide induced liver cirrhosis in rats. Both APOX and GPOX showed marked reduction in their activities in all EAF treated rats. These antioxidant enzymes are known for their beneficial role in neutralizing peroxides generated during oxidative metabolism. Decrease in the activity of both enzymes as compared to untreated control might be due to lesser production of peroxides due to the protective services extended by other antioxidative enzymes. The GSH is known for its role in glutathione redox cycle where they extend their protective function against oxidation of cellular proteins [44]. Decreased level of GSH suggests the cause of reduction in protein level in DMBA treated rats. Oxidative damage to cellular proteins might caused the reduction in protein content while groups showing increased GSH level also showed a corresponding increase in protein content. GR activity was decreased in DMBA treated rats suggesting an inability to reduce its oxidized glutathione. Highest dose of EAF, showed increased activity of GR suggesting its role in maintaining the basal level of GSH. Combined activities of GSH and GR help the affected organ to come out of oxidative damage.

LDH is a marker enzyme of cellular damage. An increased activity suggests the hepatic necrosis which in turn leads to the leakage of enzymes [45]. Raised LDH activity in DMBA treated rats in liver homogenate reflects DMBA induced hepatic injury. A dose dependent lowering of LDH activity in EAF treated rats might be due to counterworking of EAF against DMBA induced damage. Higher dose of EAF, even reduced the LDH activity below what was observed in untreated control. This might hint towards the hepatoprotective nature of EAF.

Oxidative deterioration of lipids follows successive breakdown of carbon-carbon double bonds into a number of breakdown products like conjugated dienes, lipid hydroperoxides and lipid peroxides. Treatment of DMBA alone increased the level of conjugated dienes, lipid hydroperoxides and lipid peroxides in comparison to untreated control showing increased oxidative damage operating in group II rats. Hepatoprotective nature of I3C was proved by marked reduction in lipid degradation products. Similar pattern of decrement was observed in all the treatments of EAF. This decrease in oxidative damage to liver shows the hepatoprotective nature of EAF. Similar results were obtained by Kamel and Morsy [46] where they found increased MDA content in group of rats treated with carbon tetrachloride.

Both GOT and GPT transaminases are liver enzymes and play substantive role in protein metabolism. An increase in the level of these enzymes in serum indicates liver damage. DMBA treated rats showed highest activity of both enzymes. A significant reduction in their levels with treatment of EAF hints toward hepatoprotective role of EAF. Similar results were obtained in ALP enzyme. Elevated activity of transaminases and ALP might be due to the liver damage which in turn could have allowed seepage of these enzymes from cytoplasm of liver cells into blood. Similar results were obtained by Singh et al. [8] where they induced hepatic damage by DMBA and showed increased activity of liver marker enzymes.

The UHPLC analysis of ethyl acetate fraction showed the presence of gallic acid, catechin, chlorogenic acid, epicatechin, caffeic acid, umbelliferone, coumaric acid, rutin, ellagic acid, quercetin and kaempferol polyphenols, most of which are reported to be hepatoprotective in various models of hepatic injury in rodents. The observed effect of ethyl acetate fraction on phase I, II and antioxidant enzymes may be due to the cocktail of polyphenolic compounds. Further studies are required to investigate possible synergistic effect of various compounds present in EAF as well as their molecular mechanism operating against DMBA induced hepatic damage.

## Conclusion

**Figure 7:**
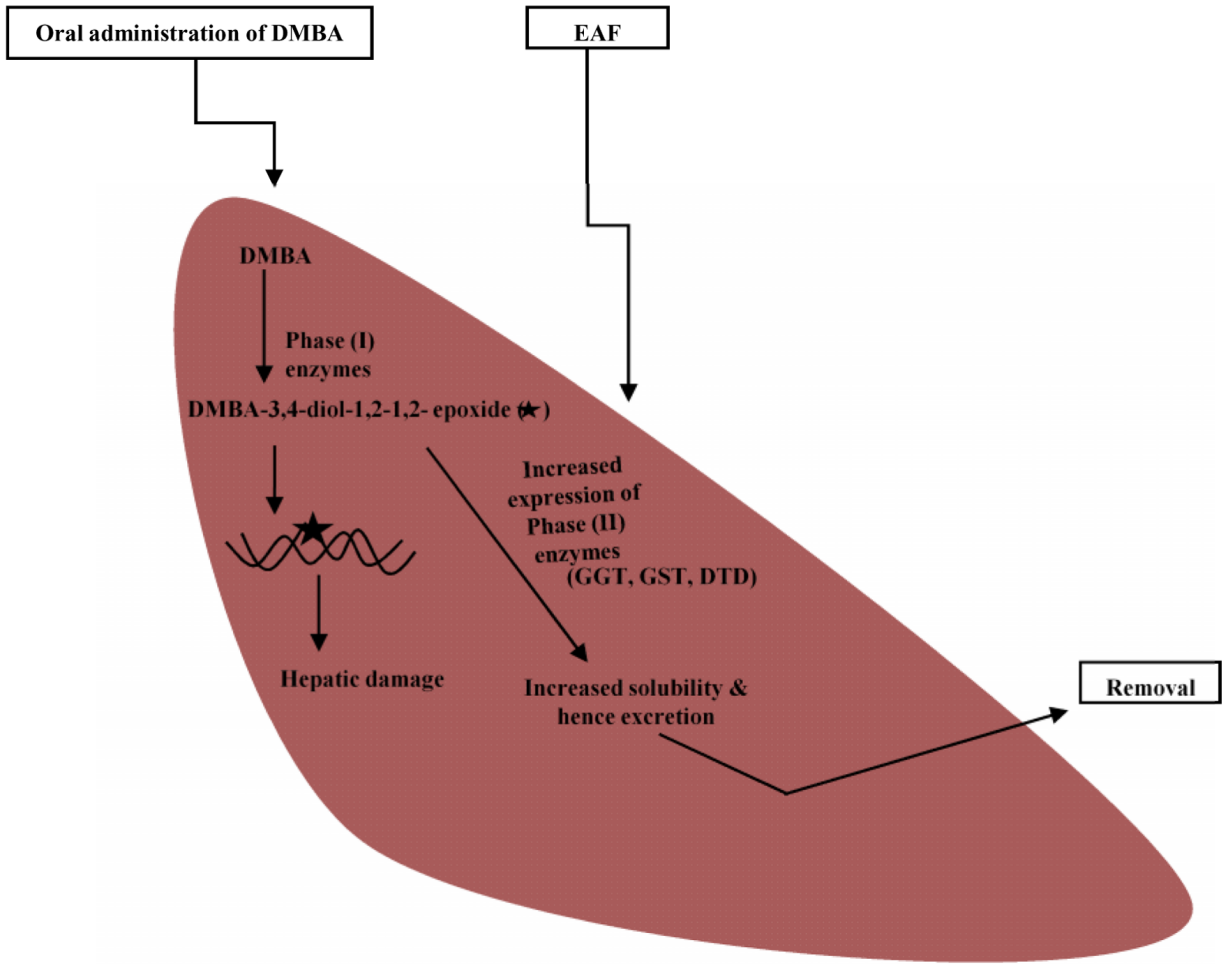
Proposed functioning of Ethyl acetate fraction (EAF) as hepatoprotective agent by upregulating phase (II) enzymes against 7,12-dimethylbenz(α)anthracene (DMBA) induced hepatocarcinogenesis.

Hence, it is concluded that ethyl acetate fraction of *Acacia catechu* Willd. as potential hepatoprotective candidate which exerted its protective effect by modulating the antioxidant, phase I and phase II enzymes (Figure 7).
